# Alpha lipoic acid attenuates oxidative stress-induced damage macromolecules in the brain of rats with sepsis-associated encephalopathy

**DOI:** 10.1186/cc14074

**Published:** 2014-12-03

**Authors:** LG Danielski, M Michels, D Florentino, A Viera, A Lauriano, Fabrícia Petronilho

**Affiliations:** 1 Laboratório de Fisiopatologia Clínica e Experimental (LAFICEXP), Programa de Pós, Graduação em Ciências da Saúde, Universidade do Sul de Santa Catarina (UNISUL), Tubarão, SC, Brazil

## Introduction

Pathophysiological mechanisms of sepsis-associated encephalopathy involve oxidative stress. This imbalance between the pro-oxidant and antioxidant causes damage to macromolecules such as lipids and proteins, thus the employment of antioxidants becomes an attractive proposition. Alpha lipoic acid (LA), a potent antioxidant, is able to cross the blood-brain barrier, and is an important cofactor in enzymatic and cellular energy metabolism. We aimed to determine the use of AL in oxidative damage and neutrophil infiltration in rat brain 12 and 24 hours after induction of sepsis model by cecal ligation and puncture (CLP).

## Methods

Male Wistar rats (250 to 350 g) were subjected to CLP model, with sham control. Groups were divided into sham + saline, sham + AL, CLP + saline and CLP + AL (200 mg/kg orally with single administration after CLP), *n *= 10. At 12 and 24 hours, rats were euthanized, the hippocampus, striatum, cerebellum, cortex and prefrontal cortex removed, lipid peroxidation assessed by TBARS, damage to proteins by protein carbonylation, myeloperoxidase activity (MPO) and the formation of nitrite and nitrate. Data were analyzed by ANOVA with *post hoc *Tukey test and log-rank test with *P *< 0.05.

## Results

In 12 hours compared with the CLP group, the CLP + AL group showed a reduction in lipid peroxidation in the striatum, in the protein carbonylation in the cortex and hippocampus, in the MPO activity in the striatum and hippocampus, and decreased formation of nitrite and nitrate in the hippocampus and cortex.

## Conclusion

While differences were not observed in 24 hours, TBARS found protein carbonylation in a reduction of damage to the CLP + AL group over the cerebellum, MPO in the striatum, hippocampus and prefrontal, and hippocampus, cerebellum, and prefrontal to nitrite/nitrate. AL may be an important therapeutic target in reducing neurologic complications in animal models of sepsis.

